# aPKC Phosphorylation of Bazooka Defines the Apical/Lateral Border in *Drosophila* Epithelial Cells

**DOI:** 10.1016/j.cell.2010.02.040

**Published:** 2010-04-30

**Authors:** Eurico Morais-de-Sá, Vincent Mirouse, Daniel St Johnston

**Affiliations:** 1 The Gurdon Institute and the Department of Genetics, University of Cambridge, Tennis Court Road, Cambridge CB2 1QN, UK

**Keywords:** DEVBIO, CELLBIO

## Abstract

Bazooka (PAR-3), PAR-6, and aPKC form a complex that plays a key role in the polarization of many cell types. In epithelial cells, however, Bazooka localizes below PAR-6 and aPKC at the apical/lateral junction. Here, we show that Baz is excluded from the apical aPKC domain in epithelia by aPKC phosphorylation, which disrupts the Baz/aPKC interaction. Removal of Baz from the complex is epithelial-specific because it also requires the Crumbs complex, which prevents the Baz/PAR-6 interaction. In the absence of Crumbs or aPKC phosphorylation of Baz, mislocalized Baz recruits adherens junction components apically, leading to a loss of the apical domain and an expansion of lateral. Thus, apical exclusion of Baz by Crumbs and aPKC defines the apical/lateral border. Although Baz acts as an aPKC targeting and specificity factor in nonepithelial cells, our results reveal that it performs a complementary function in positioning the adherens junction in epithelia.

## Graphical abstract

**Graphical Abstract:**

**Figure:**
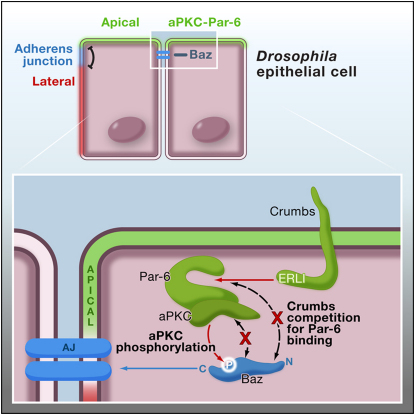

**Highlights:**

► aPKC phosphorylates Bazooka to prevent its binding to aPKC ► Crumbs outcompetes phospho-Baz for binding to PAR-6 in epithelial cells ► This mechanism defines the apicial/lateral boundary in epithelia ► Failure to exclude Baz from the aPKC complex disrupts epithelial morphogenesis

## Introduction

A key step in the generation of cell polarity is the localization of conserved cortical polarity complexes to opposite sides of the cell, where they regulate many polarized aspects of cell behavior, such as membrane trafficking and the organization of the cytoskeleton. The polarity of all polarized cell types investigated so far depends on the PAR-3 or Bazooka (Baz) complex, which comprises the multiple PDZ domain protein, PAR-3 (Baz in *Drosophila*), the semi-CRIB and PDZ domain protein, PAR-6, and atypical Protein Kinase C (aPKC) (Goldstein and Macara, 2007).

The PAR-3 complex was first identified in the *C. elegans* zygote, where it localizes to the anterior cortex, with PAR-2 and PAR-1 forming a complementary posterior cortical domain (Etemad-Moghadam et al., 1995; Hung and Kemphues, 1999; Tabuse et al., 1998). The PAR protein asymmetry directs the localization of cytoplasmic determinants and the orientation of the first mitotic spindle, resulting in an asymmetric cell division that generates the anterior-posterior (AP) axis of the worm (Gönczy, 2008; Siller and Doe, 2009). The PAR proteins play a similar role in the formation of the AP axis in *Drosophila*. At stage 9 of oogenesis, PAR-1 and Lgl localize to the posterior cortex of the oocyte, and Baz (PAR-3), PAR-6, and aPKC mark the anterior and lateral cortex (González-Reyes et al., 1995; Roth et al., 1995; Shulman et al., 2000; Tian and Deng, 2008). This polarized distribution of PAR proteins induces the formation of an anterior-posterior gradient of microtubules that directs the localization of the axis determining transcripts to define the AP axis of the future embryo (Bastock and St Johnston, 2008).

The Baz/Par-6/aPKC complex also plays an essential role in polarizing the asymmetric divisions of the *Drosophila* neuroblasts (Gönczy, 2008; Siller and Doe, 2009). As the neuroblast enters mitosis, Baz recruits PAR-6/aPKC to the apical cortex, and aPKC then phosphorylates Numb and Miranda to exclude them from the apical region, thereby localizing the basal determinants (Atwood and Prehoda, 2009; Wirtz-Peitz et al., 2008). Baz binds directly to Numb to recruit it for aPKC phosphorylation, and therefore functions both as a localization factor and substrate specificity determinant for aPKC in the polarization of the neuroblast division.

Epithelia form the majority of tissues in the body, and must be polarized along their apical-basal axis to perform their essential functions as barriers between different compartments. Unlike the *C. elegans* zygote and the *Drosophila* oocyte and neuroblast, epithelial cells have at least four distinct cortical domains: an apical domain, an apical-lateral junction (the tight junction in vertebrates and the Adherens junction [AJ] in *Drosophila*), a lateral domain, and a basal domain. The formation of the apical-lateral junction is key feature of epithelia, as it holds adjacent cells together to form epithelial sheets, provides the barrier to paracellular diffusion in mammals, and demarcates the boundary between apical and basolateral membrane domains (Anderson et al., 2004).

As in other cell types, Baz/PAR-3, PAR-6, and aPKC are essential for the formation of polarized epithelia (Goldstein and Macara, 2007). In addition to the PAR proteins, epithelial polarity depends on the Crumbs (Crb) and “Scribble” polarity complexes. The Crb complex contains the transmembrane protein, Crb, the MAGUK protein, Stardust/PALS1 (Sdt), and Patj (Assémat et al., 2008). This complex localizes to the apical domain in both *Drosophila* and mammalian epithelia and seems to act as the apical determinant (Lemmers et al., 2004; Roh et al., 2003; Tepass et al., 1990; Wodarz et al., 1995). By contrast, the components of the Scribble complex, Scribble, Dlg, and Lgl, localize below the apical-lateral junction, where they antagonize the Crb complex (Bilder et al., 2003; Tanentzapf and Tepass, 2003).

Although Baz/PAR-3, PAR-6, and aPKC are often assumed to function as a complex in epithelial cells, there is increasing evidence that Baz/PAR-3 acts independently from PAR-6 and aPKC in this cell type. First, PAR-6 and aPKC localize to the apical and subapical region in many different epithelia, whereas most Baz/PAR-3 is localized slightly more basally, at the level of the AJs in flies and the tight junctions in vertebrates (Afonso and Henrique, 2006; Harris and Peifer, 2005; Martin-Belmonte et al., 2007; Satohisa et al., 2005). Second, PAR-6 and aPKC interact with the Crb complex. Both Sdt and Crb can bind directly to the PDZ domain of PAR-6, and they coprecipitate with PAR-6 and aPKC in mammals and *Drosophila* (Hurd et al., 2003; Kempkens et al., 2006; Lemmers et al., 2004; Nam and Choi, 2006; Wang et al., 2004a). Furthermore, two conserved threonines in the cytoplasmic tail of Crb are phosphorylated by aPKC, and this is required for Crb activity (Sotillos et al., 2004). Baz/PAR-3, on the other hand, interacts with components of the apical junction in both flies and mammals. In *Drosophila*, Baz interacts with Armadillo (Arm), which binds directly to *D*E-Cadherin, as well as the Nectin-like protein, Echinoid, both of which are components of the AJs, while mammalian PAR-3 binds to the tight-junction proteins JAM1-3 and Nectin (Ebnet et al., 2001; Itoh et al., 2001; Takekuni et al., 2003; Wei et al., 2005). Indeed, Baz plays a key role in positioning the AJs in the primary epithelium of *Drosophila*, as it localizes to the apical/lateral border before Cadherin and Arm, and is required for the coalescence of spot AJs into the zonula adherens (Harris and Peifer, 2005; McGill et al., 2009). PAR-3 plays a similar role in the formation of tight junctions in repolarising MDCK cells, and this depends on its interaction with the Rac exchange factor, TIAM1, but is independent of binding to aPKC (Chen and Macara, 2005).

The fact that Baz (PAR-3) and PAR-6/aPKC seem to function in different complexes in epithelia raises the question of why Baz does not colocalize with PAR-6 and aPKC, when it can bind directly to both of them. Baz /PAR-3 interacts with the kinase domain of aPKC through its third conserved region (CR3), and PAR-3 has been shown to bind the PDZ domain of PAR-6 through its first PDZ domain (Izumi et al., 1998; Joberty et al., 2000; Lin et al., 2000). We therefore set out to determine the mechanisms that exclude Baz from the apical aPKC/PAR-6 domain and to investigate whether this is important for the establishment of epithelial polarity in *Drosophila*.

## Results

### Baz Localizes below PAR-6 and aPKC in the Follicular Epithelium

**Figure 1:**
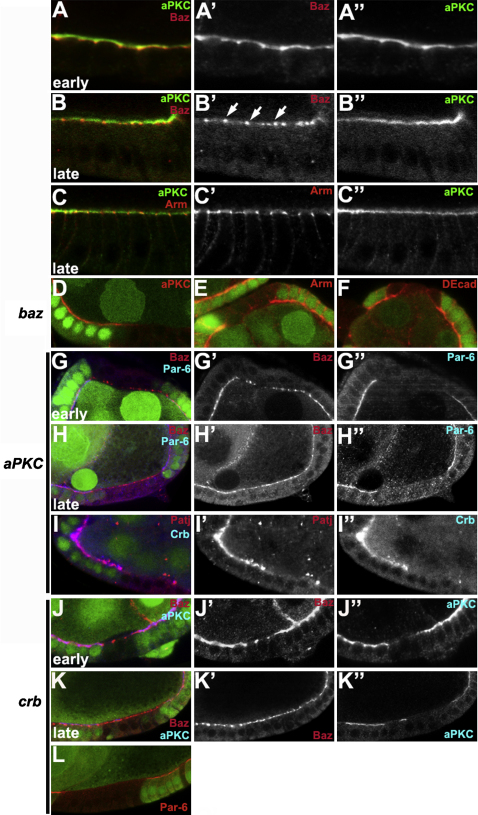
Bazooka Localizes below Par-6/aPKC in the Follicle Cells (A and B) Baz (red) and aPKC (green) staining in cuboidal (A) and columnar follicle cells (B; arrows show Baz localization at the junctions). (C) aPKC (green) and Arm (red) staining in columnar follicle cells. (D–L) Mosaic egg chambers containing mutant follicle cell clones marked by the loss of nuclear GFP (green). (D–F) *baz^4^* follicle cell clones stained in red for aPKC (D), Arm (E), and E-Cadherin (F). (G and H) *aPKC^K06403^* follicle cell clones stained for Baz (red and G′ and H′) and PAR-6 (blue and G″ and H″). (I) *aPKC^K06403^* follicle cell clones stained for Patj (red and I′) and Crb (blue and I″). (J and K) *crb^2^* clones in cuboidal (J) and columnar follicle cells (K) stained for Baz (red and J′ and K′) and aPKC (blue, J″ and K″). (L) *crb^2^* follicle cell clones stained for PAR-6 (red).

We first examined the localization of Baz and aPKC relative to each other and the AJs in the follicle cells that surround the developing germline cysts of the *Drosophila* ovary, since these cells form a polarized monolayer that is easily imaged along the apical-basal axis. aPKC localizes to the apical domain of the follicle cells throughout oogenesis (Figures 1A–1C). Baz localizes slightly more basally than aPKC with a partial overlap in early oogenesis when the follicle cells are cuboidal and is enriched at the apical/lateral junctions (Figure 1A). This difference is more marked once the cells have become columnar at stage 9, when almost all Baz localizes to the AJs with Arm (Figures 1B and 1C).

The different positions of Baz and aPKC are reflected in the distinct genetic requirements for the localization of each protein. Removal of Baz abolishes the apical localization of aPKC and PAR-6 at all stages and blocks AJ formation, as seen by the lack of Arm and DE-Cadherin localization in mutant cells (Figures 1D–1F and data not shown) (Abdelilah-Seyfried et al., 2003). By contrast, Baz localization is largely independent of aPKC and PAR-6. Cuboidal follicle cells mutant for *aPKC* and *par-6* lack the most apical pool of Baz, but the junctional pool is largely unaffected (Figure 1G′ and data not shown). Furthermore, *aPKC* mutant columnar follicle cells have a wild-type distribution of Baz, even though PAR-6 is unlocalized (Figure 1H). Thus, Baz localizes in the follicle cells independently of aPKC, whereas aPKC localization requires Baz. This is similar to the primary epithelium of embryo, where Baz localizes to the apical/lateral cortex earlier than and independently from aPKC and Par-6, which subsequently localize more apically (Harris and Peifer, 2005). These results reinforce the view that Baz and PAR-6/aPKC form distinct complexes in epithelia.

We also examined the relationship between aPKC and the Crb complex. Loss of aPKC abolishes the localization of Crb and Patj, and loss of Crb disrupts the localization of aPKC and PAR-6 (Figures 1I–1L). Baz is still localized in *crb* null mutant cells, however, although the most apical pool of the protein is lost in cuboidal cells, as it is in *aPKC* mutants (Figures 1J and 1K). These results suggest that PAR-6 and aPKC associate with the Crb complex, whereas Baz is primarily associated with the junctions.

### aPKC Phosphorylates Bazooka to Exclude It from the Apical Domain

**Figure 2:**
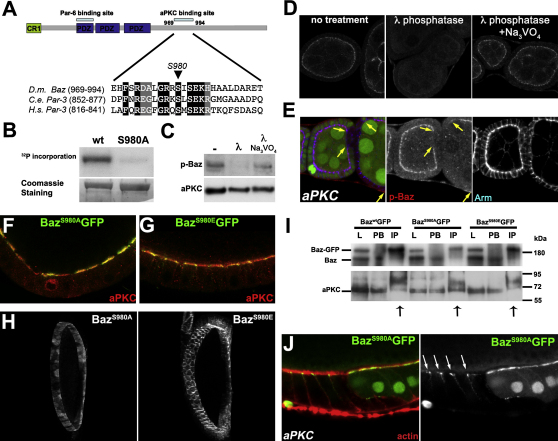
aPKC Phosphorylates Bazooka to Exclude it from the Apical Domain (A) Diagram showing the structure of Baz with the position of the PAR-6 and aPKC binding sites. An alignment of the aPKC binding sites of *Drosophila* Baz (*D.m.*), *C.elegans* (*C.e.*), and *H.sapiens* (*H.s.*) PAR-3 is shown below. (B) aPKC was coimmunoprecipitated from PAR-6-GFP-expressing embryos and incubated with recombinant MBP:Baz (amino acids 829–1168) or MBP:Baz^S980A^ in presence of [γ-^32^P] ATP. Radioactive proteins were detected by SDS-PAGE followed by autoradiography. The loading control is shown. (C) Western blot of ovary extracts probed with an α-phospho-BazS980 antibody. −, no treatment; λ, lambda phosphatase treatment; λ + Na_3_VO_4_, lambda phosphatase and a phosphatase inhibitor. The blot was reprobed with α-aPKC as a loading control. (D) Phospho-Baz staining in the follicular epithelium. (E) *aPKC^K06403^* follicle cell clones (delimited by arrows) stained for phospho-Baz (red) and Arm (blue). (F and G) Localization of GFP-tagged forms of Baz^S980A^ (F) and Baz^S980E^ (G, green). aPKC staining is shown in red. (H) Projections of Z stacks showing the distributions of Baz^S980A^:GFP and Baz^S980E^:GFP in stage 11 egg chambers. (I) Immunoprecipitation of Baz:GFP with anti-GFP antibody from extracts of ovaries expressing different Baz constructs. One-one hundredth of the input lysate (L), 1/100 of the lysate after IP (PB), and the immunoprecipitated fraction (IP) were immunoblotted with α-Baz or α-aPKC antibodies. Much more aPKC immunoprecipitates with Baz^S980A^:GFP (arrows). The bands above the aPKC band in the IP fraction result from the use of IgG-coupled beads. (J) An *aPKC ^K06403^* follicle cell clone expressing Baz^S980A^:GFP (green) and stained for actin (red). Unphosphorylatable Bazooka is found at the apical-lateral junctions (arrows) in *aPKC* mutant cells (marked by loss of nuclear GFP). See also Figure S1.

Mammalian aPKC phosphorylates PAR-3 on a serine within its aPKC binding site to destabilize the PAR-3/aPKC interaction (Nagai-Tamai et al., 2002). This site is conserved in Baz (Figure 2A), suggesting that the regulation of Baz by aPKC phosphorylation may be conserved. To test this, we examined whether *Drosophila* aPKC can phosphorylate Baz on S980. Since the substrate specificity of aPKC depends on its binding partners, we purified native *Drosophila* aPKC from embryos by coimmunoprecipitating it with PAR-6:GFP. Purified aPKC phosphorylated the region of Baz containing the aPKC-binding site, but did not phosphorylate the equivalent region with the S980A mutation (Figure 2B).

**Figure S1:**
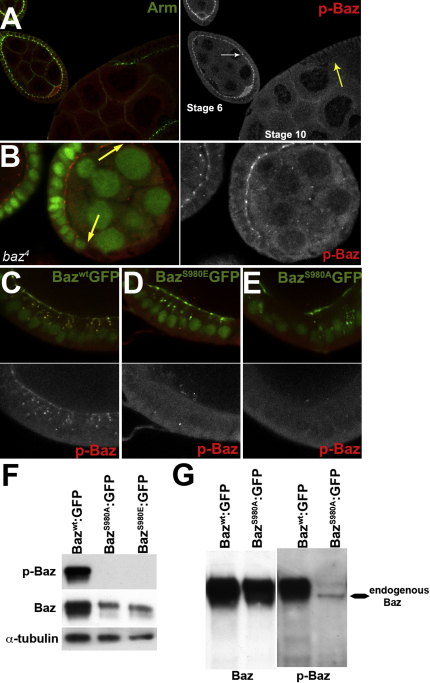
Specificity of p-Baz Antibody for S980 Phosphorylation, Related to Figure 2 (A) A stage 6 and an early stage 10 egg chamber stained for Armadillo (green) and phospho-Baz (red and right panel). The levels of phospho-Baz are much lower in the columnar follicle cells that have completed their posterior migration (yellow arrow). (B) A *baz*^4^ mutant clone (delimited by arrows) marked by the loss of nuclear GFP (green) stained for phospho-Baz (red and right panel). No phospho-Baz signal is observed in the mutant cells, confirming the specificity of the antibody. (C-E) Baz^WT^:GFP, Baz^S980E^:GFP and Baz^S980A^:GFP were co-expressed with nuclear GFP in columnar follicle cells under the control of AyGal4. The α-phospho Baz antibody (red and lower panels) recognizes Baz^WT^:GFP, but not Baz^S980A^:GFP or Baz^S980E^:GFP fusions. (F and G) Western blots of extracts from 2-5h embryos expressing the indicated Baz transgenes under the control of matα4GAL4VP16. The same membrane was probed for phospho-Baz, Baz and α-tubulin as loading control. (F) The anti-phospho-Baz antibody does not recognize the Baz variants that lack S980. (G) Over-exposure of the membrane reveals the specificity of the antibody, and a band for endogenous phosphorylated Baz in the Baz^S980A^:GFP overexpression extracts. This band is masked in the Baz^WT^:GFP lane by the strong staining of the phosphorylated transgenic protein.

To confirm that aPKC phosphorylates Baz in vivo, we generated phospho-specific antibodies against BazS980-P. These recognized a single band on western blots of ovary extracts, which was strongly reduced after treatment with λ-phosphatase, confirming that the antibodies are specific for BazS980-P and that this site is phosphorylated in vivo (Figure 2C). Furthermore, the P-Baz antibodies recognized wild-type Baz fused to GFP in embryos and ovaries, but not a S980 mutant, further demonstrating their specificity for BazS980-P (Figure S1 available online). The phospho-specific antibodies also specifically detect BazS980-P at the apical side of follicle cells, since no staining is observed after phosphatase treatment or in *baz* mutant clones (Figure 2D and Figure S1B). BazS980-P staining was abolished in *aPKC* mutant follicle cell clones, indicating that it is the result of aPKC activity (Figure 2E). Thus, aPKC directly phosphorylates Baz on S980 in vivo at the apical side of the follicle epithelium. Phosphorylation is developmentally regulated, as much lower levels are seen once the cells have undergone the cuboidal to columnar transition (Figure S1A).

To address whether the localization of Baz is regulated by aPKC phosphorylation, we expressed nonphosphorylatable (Baz^S980A^) and phosphomimetic (Baz^S980E^) versions of Baz fused to GFP. Wild-type Baz-GFP shows an identical localization to the untagged protein, with most localized to the AJs. By contrast, Baz^S980A^ localizes to the apical domain with aPKC, whereas Baz^S980E^ concentrates at the AJs (Figures 2F and 2G). The complementary patterns of unphosphorylatable and phosphomimetic Baz are particularly clear in projections: the former forms a cap at the apical side of each cell, whereas the latter forms a lattice around the apical-lateral margins (Figure 2H).

These observations indicate that aPKC phosphorylation controls the distribution of Baz: BazS980E, which cannot bind aPKC, is restricted to the apical-lateral border, whereas BazS980A is recruited to the apical domain, presumably by binding to aPKC. Consistent with this, much more aPKC coimmunoprecipitates with Baz^S980A^-GFP than with wild-type Baz or Baz^S980E^ from ovary extracts (Figure 2I). To test more directly whether the apical localization of Baz^S980A^ depends on binding to aPKC, we examined its localization in *aPKC* mutant clones. Baz^S980A^ no longer localizes apically in mutant cells, and accumulates instead at the apical-lateral boundary (Figure 2J). Thus, phosphorylation by aPKC excludes Baz from the apical domain by preventing its association with aPKC itself.

### aPKC Phosphorylation of Bazooka Is Essential for Epithelial Organization

**Figure 3:**
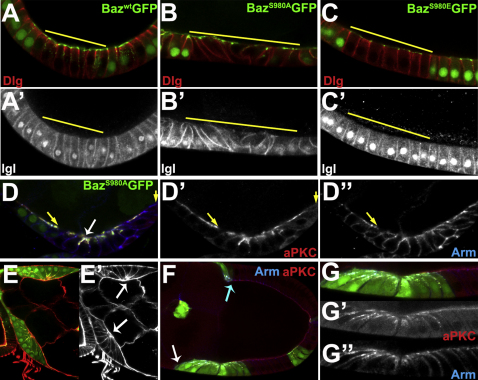
aPKC Phosphorylation of Bazooka Is Essential for Epithelial Organization (A–D) *baz^4^* follicle cell clones (yellow lines) expressing Baz^WT^:GFP (A), Baz^S980A^:GFP (B and D), or Baz^S980E^:GFP (C) stained for Dlg (red in A–C), Lgl (A′–C′), aPKC (red in D and D′) and Arm (blue in D and D″). The arrow in (D) indicates the formation of apical constrictions in Baz^S980A^-rescued cells. (E) Overexpression of Baz^S980A^:GFP in Flpout clones marked by GFP expression (green). F-actin is stained in red (shown in E′). Baz^S980A^ overexpression induces apical constriction (arrows). (F) A stage 9 egg chamber containing clones of Baz^S980A^:GFP-overexpressing cells (green) stained for aPKC (red) and Arm (blue). The large clone of Baz^S980A^-expressing cells fails to migrate normally (compare white arrow with blue arrow). (G) A close up of a clone of Baz^S980A^-expressing cells in (F) showing aPKC (G′) and Arm (G″).

To investigate the functional importance of aPKC phosphorylation of Baz, we tested whether the different Baz transgenes could rescue the *baz* null phenotype. We first expressed the transgenes with e22cGal4, a constitutive epithelial driver, but BazS980A:GFP was lethal under these conditions. We therefore induced FLPout *baz* mutant clones in the follicular epithelium in which the different Baz constructs were expressed with Ay-Gal4. *baz* mutant follicle cells lose their apical-basal polarity and form multiple layers or leave the epithelium entirely, resulting in gaps in the epithelial covering of the germline cyst (Benton and St Johnston, 2003a). Wild-type Baz-GFP rescues this phenotype completely (Figure 3A). Expression of Baz^S980E^-GFP also rescued the *baz* mutant phenotype, indicating that the interaction between Baz and aPKC is dispensable for normal apical-basal polarity in the follicular epithelium (92% wild-type morphology and aPKC localization, n = 71; Figure 3C).

We recovered large *baz* mutant clones expressing Baz^S980A^-GFP only rarely, and the resulting egg chambers often had large gaps in the follicle cell layer, suggesting that mutant cells fail to integrate into the epithelium. We recovered many small clones induced after the epithelium had formed, but the *baz*, Baz^S980A^-GFP mutant cells were usually disorganized with aberrant cell shapes (83%, n = 65; Figure 3B). Most Baz^S980A^-expressing mutant cells underwent apical constriction to become wedge shaped, and Arm was often mislocalized apically, where it overlapped with aPKC (Figure 3D). This phenotype suggests that Baz phosphorylation by aPKC is required to establish the boundary between the apical and junctional domains, since AJ proteins localize apically in its absence. *baz* mutant cells expressing Baz^S980A^-GFP did retain some apical-basal polarity, as the lateral markers, Lgl and Dlg, were still excluded from the apical domain (Figures 3B and 3B′). This shows that Baz^S980A^ rescues some Baz functions, and does not disrupt the epithelium by acting as an inhibitor of aPKC's kinase activity, as aPKC activity is required to exclude Lgl from the apical domain.

Since Baz^S980A^-GFP is mislocalized to the apical domain, we also asked whether it produced a gain-of-function phenotype when overexpressed with the FLPout/tub-GAL4 system. While wild-type Baz and Baz^S980E^ had no effect under these conditions, many Baz^S980A^-expressing cells showed a marked constriction of their apical surfaces, which caused the apical sides of the cells to cluster (Figures 3E and 3E′). In addition, Baz^S980A^ expression delayed the cuboidal to columnar transition: clones in the anterior region of the epithelium were usually shorter than their neighbors and were inhibited in their posterior movement to envelop the oocyte (Figure 3F). Finally, Baz^S980A^ expression induced the apical localization of Arm with both Baz^S980A^-GFP and aPKC (Figures 3G–3G″. This suggests that Baz^S980A^ mislocalizes to the apical domain because it binds aPKC, and then recruits Arm and Cadherin. The apical constriction might then occur because the mislocalized Cadherins in adjacent Baz^S980A^-expressing cells adhere to each other, leading to an apical extension of the AJs that reduces the apical surface area and expands the lateral domain.

### The Role of Baz Phosphorylation in the Embryo

**Figure 4:**
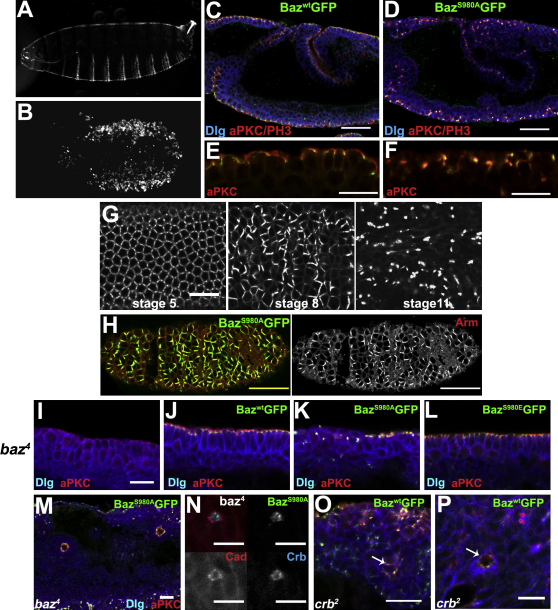
aPKC Phosphorylation of Baz Is Required in Embryonic Epithelia (A–H) Wild-type embryos overexpressing Baz^WT^:GFP (A, C, and E) or Baz^S980A^:GFP (B, D, F, G, and H) under the control of matα4Gal4-VP16. (A and B) Dark-field images of cuticle preparations of embryos expressing Baz^WT^:GFP (A) or Baz^S980A^:GFP (B). (C and D) Stage 9 embryo expressing Baz^WT^:GFP (green) (C) or Baz^S980A^:GFP (D) stained for aPKC (red), Phospho-Histone 3 (red), and Dlg (blue). (E and F) Cross-sections of regions of the epidermis of stage 9 embryos expressing Baz^WT^:GFP (E) or Baz^S980A^:GFP (F) stained for aPKC (red). (G) Images from fixed Baz^S980A^:GFP embryos at progressive stages of development. (H) Arm (red and as separate channel) aggregates with Baz^S980A^:GFP (green). (I–M) *baz*^4^ mutant embryos with no transgene (I), or with zygotic expression of Baz^WT^:GFP (J), Baz^S980A^:GFP (K and M), or Baz^S980E^:GFP (L) stained for aPKC (red) and Dlg (blue). (N) Close up of a baz^4^, Baz^S980A^:GFP rescued cyst stained for E-Cadherin (red) and Crb (blue). (O) A stage 12 *crb^2^* embryo expressing Baz^WT^:GFP and stained for aPKC (red) and Dlg (blue). Baz^WT^:GFP colocalizes with aPKC in aggregates in the disorganized epithelium, which is forming epithelial cysts. (P) Close up of an epithelial cyst in a *crb^2^* mutant embryo. The scale bars represent 50 μm in (C), (D), and (H), 20 μm in (E)–(G), (I), (M), and (O), and 10 μm in (N) and (P). See also Figure S1 and Movies S1 and S2.

**Figure S2:**
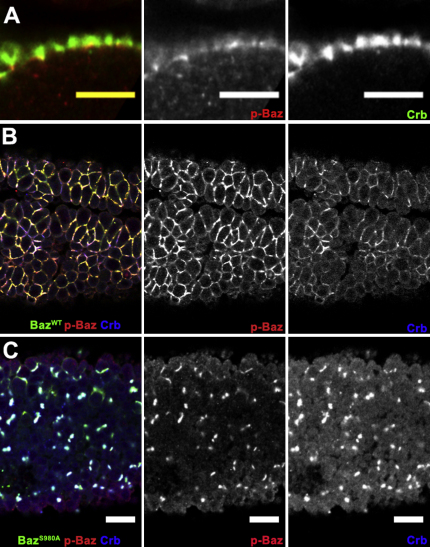
Baz Phosphorylation in the Embryonic Epithelium, Related to Figure 4 (A) Cross section of the embryonic epithelium showing that phospho-Baz (red) accumulates basally to Crb (green). (B and C) Ventral surface sections of embryos with maternal overexpression of Baz^WT^:GFP and Baz^S980A^:GFP stained for phospho-Baz (red) and Crb (blue). Note that endogenous phosphorylated Baz and Crb accumulate in junctional aggregates in Baz^S980A^:GFP overexpressing embryos. Scale bar (A) 10 μm (C) 20 μm.

Baz and aPKC also have distinct localizations and functions during the formation of the primary epithelium of the embryo, raising the possibility that Baz phosphorylation also plays a role in this tissue. Indeed, Baz is phosphorylated in the forming embryonic epithelium and localizes beneath the Crb/aPKC domain (Figure S2A). Maternal overexpression of wild-type Baz-GFP has no effect on embryogenesis, and the resulting larvae have normal cuticles (Figure 4A). By contrast, expression of Baz^S980A^ at lower levels causes embryos to die with disorganized epithelia that secrete small grains of cuticle, a phenotype that closely resembles that of *crumbs* mutants (Figure 4B and Figure S1F). Epithelial organization is already severely disrupted by stage 9 of embryogenesis, although the major morphogenetic events still occur (Figures 4C and 4D).

The majority of Baz localizes below aPKC in wild-type and Baz^WT^:GFP-expressing embryos at stage 9 (Figures 4E) (Harris and Peifer, 2005). By contrast, Baz^S980A^:GFP colocalizes with aPKC to form large aggregates that also contain the junctional components Arm and E-Cadherin and the apical components PAR-6, Crb and Patj (Figures 4F and 4H, Figure S2C, and data not shown). 3D reconstructions reveal that Baz^S980A^:GFP eventually induces the formation of a single large aggregate in each epithelial cell, instead of the typical hexagonal pattern of Baz^WT^:GFP (Movie S1). Phospho-Baz also accumulates in these junctional aggregates, indicating that endogenous Baz is recruited to the aggregates, presumably by dimerizing with BazS980A through its N-terminal CR1 domain (Benton and St Johnston, 2003a) (Figure S2B).

We performed time-lapse imaging to identify exactly how Baz^S980A^:GFP-expressing embryos lose their epithelial integrity (Movie S2). These embryos cellularize normally, but after gastrulation Baz^S980A^:GFP starts to accumulate at random cell-cell junctions, which then gradually coalesce into a single aggregate per cell (Figure 4G). Arm colocalizes with Baz^S980A^ throughout this process, suggesting that the AJs also collapse into a single junction in each cell. These aggregates start to form during the fast phase of germband elongation, when the ectoderm extends as a result of cell intercalation and cell shape changes (Movie S2). However, aggregates form in all epithelial structures, including those in the head, indicating that Baz^S980A^:GFP disrupts epithelial organization in general. (Movie S2 and Figure 4H).

We also analyzed whether the Baz variants could rescue the phenotype of *baz^4^* zygotic mutants by expressing them zygotically from paternally derived transgenes (zygotic expression of Baz^S980A^:GFP does not cause a dominant phenotype because it is expressed later and at lower levels than when contributed maternally). Unlike *baz* maternal/zygotic mutants, which show defects during cellularization, *baz^4^* zygotic mutants still contain maternally loaded Baz, which allows them to form normal epithelia until stage 11–13 of embryogenesis (Harris and Peifer, 2005; Tanentzapf and Tepass, 2003). The maternal pool of Baz runs out at this stage, however, and the epithelia become disorganized and fail to maintain the apical localization of aPKC (Figure 4I). Both Baz^WT^:GFP and Baz^S980E^:GFP rescue aPKC localization and epithelial organization in *baz* null mutants (Figures 4J and 4L). Baz^S980A^:GFP, on the other hand, does not rescue *baz*^4^, and the mutant embryos have disorganized epithelia, in which Baz^S980A^ and aPKC colocalize in randomly positioned junctions in most of the ectoderm (Figure 4K). By the end of embryogenesis, these embryos form epithelial cysts with internal apical domains that contain Baz^S980A^:GFP, Crb and Cadherin (Figures 4M and 4N). The terminal phenotype of *baz*^4^, Baz^S980A^:GFP embryos is reminiscent of that of *crb* and *sdt* mutants (Bilder et al., 2003; Tanentzapf and Tepass, 2003). For example, *crb* mutants develop fragmented junctions, in which aPKC and Baz^WT^:GFP colocalize (Figure 4O). Furthermore, at the end of embryogenesis, *crb* embryos form polarized cyst-like structures with internal apical domains with colocalized Baz and aPKC (Figure 4P). Thus, Baz^WT^:GFP in a *crb* mutant behaves like Baz^S980A^, in that it is not excluded from the apical aPKC domain, supporting the view that Crb is necessary for the segregation of Baz from aPKC (Harris and Peifer, 2005).

### Crumbs Function in the Apical Exclusion of Baz Is Independent of aPKC Phosphorylation

**Figure 5:**
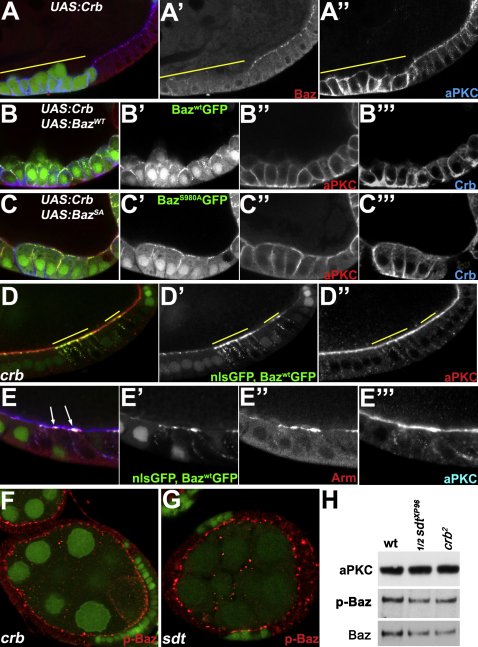
Crumbs Is Required for the Apical Exclusion of Bazooka Independently of aPKC Phosphorylation (A) Flpout clones overexpressing UAS-Crb and UAS-GFP (green, yellow line) stained for Baz (red and A′) and aPKC (blue and A″). Crb causes the cells to lose polarity and recruits aPKC around the entire cell cortex. (B and C) Flpout clones overexpressing UAS-Crb, UAS-GFP (green, B′, and C′), and UAS-Baz^wt^:GFP (B, green, and B′) or UAS-Baz^S980A^:GFP (C, green, and C′), stained for aPKC (red, B″, and C″) and Crb (blue, B″′, and C″′). Baz^S980A^ recruits aPKC to the apical and lateral cortex and partially rescues the polarity phenotype. (D) *crb^2^* clones marked by the absence of nuclear GFP (yellow lines) expressing Baz^WT^:GFP (green) and stained for aPKC (red). (E) A close up of a *crb^2^* clone expressing Baz^WT^:GFP (green and E′) stained for Arm (red and E″) and aPKC (blue and E″′). Baz^WT^:GFP induces apical constriction (arrows). (F and G) *crb^2^* (F) and *sdt^XP96^* (G) follicle cell clones stained for phospho-Baz (red). (H) Western blot of extracts from 4–8 hr embryos probed with α-aPKC, α-phospho-Baz^S980^ and α-Baz. w- (wt), *crb^2^* maternal/zygotic embryos (*crb^2^*), and a 1:1 mixture of *sdt^XP96^* and *sdt^XP96^*/+ embryos from germline clones (1/2 *sdt^XP96^* M/Z). See also Figure S3.

**Figure S3:**
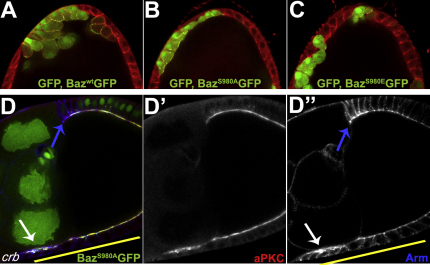
BazS980A:GFP Suppresses the Crb Overexpression Phenotype and Rescues Apical aPKC Localization in *crb* Mutants, Related to Figure 5 (A–C) Co-expression of (A) Baz^WT^:GFP (B) Baz^S980A^:GFP and (C) Baz^S980E^:GFP with Crb. Baz^WT^:GFP and Baz^S980E^:GFP co-expression do not rescue cell shape, but follicle cells co-expressing Baz^S980A^:GFP remain generally columnar. Dlg (red) labels the cell cortex. (D) Expression of Baz^S980A^:GFP with Cy2-Gal4 in *crb^2^* mosaic mutant clones marked by the absence of nuclear GFP (yellow line). Baz^S980A^:GFP rescues aPKC localization, even though rescued cells have altered epithelial morphology. Note the delay in the movement of the rescued follicle cells (white arrow) to envelop the oocyte (compared to blue arrow). Separate channels are shown next to the merged pictures.

Crb overexpression expands the apical domain and causes aPKC and PAR-6 to localize all around the cortex, whereas Baz is excluded from the cortex (Wodarz et al., 1995) (Figure 5A and data not shown). We therefore asked whether the cortical exclusion of Baz depends on its phosphorylation by aPKC, by coexpressing Baz^S980A^:GFP and Crb. Baz^S980A^ localizes to the cortex in the presence of excess Crb and is predominantly apical, with some extension along the lateral domain (Figure 5C). Although Crb still spreads around the cortex, aPKC localizes apically with Baz^S980A^ in these cells, which remain roughly columnar. Thus, Baz^S980A^ appears to out-compete Crb for binding to aPKC, and partially rescues epithelial organization. By contrast, Baz^WT^:GFP and Baz^S980E^:GFP fail to recruit aPKC when coexpressed with Crb, and the cells become unpolarized with uniform cortical Crb and aPKC (Figure 5B and Figures S3A and S3C).

To further investigate the role of Crb in Baz apical exclusion, we overexpressed Baz^WT^:GFP in *crb* mutant clones. Baz^WT^:GFP accumulates apically in *crb* mutant cells and rescues the apical localization of aPKC (Figure 5D). The Baz^WT^:GFP *crb* mutant cells also sometimes undergo apical constriction and accumulate apical Arm (Figure 5E). These effects are very similar to those of Baz^S980A^, although the latter produces this phenotype whether the cells are wild-type or *crb* mutant (Figures 3D and 3G and Figure S3D). These results reveal two important features of the relationship between Crb, aPKC, and Baz. First, they show that aPKC (and PAR-6) can localize apically when associated with either the Crb complex or Baz, which compete for PAR-6/aPKC. Second, they indicate that the apical exclusion of Baz and the formation of the apical-lateral boundary depend on both the presence of the Crb complex and aPKC phosphorylation, both of which are necessary to prevent the association of Baz with the PAR-6/aPKC complex.

Since Crb and Sdt bind to PAR-6, we hypothesized that they might activate aPKC phosphorylation of Baz, thereby triggering the removal of Baz from the PAR-6/aPKC complex. However, this does not seem to be the case. First, Baz is still phosphorylated on S980 in *crb* and *sdt* mutant follicle cell clones (Figures 5F and 5G). Second, phospho-Baz can still be detected in western blots of *crb^m/z^* embryos (Figure 5H). Although Baz levels are somewhat reduced in these *crb* null embryos, the extent of Baz phosphorylation appears unchanged. A similar result was observed in 1:1 mixture of embryos that lacked maternal *sdt* and had either one or no copies of zygotic *sdt*.

### The Crumbs Complex Disrupts the Baz/PAR-6 Interaction

The results above indicate that the Crb complex prevents Baz from associating with the PAR-6/aPKC complex independently of Baz phosphorylation by aPKC. Baz phosphorylation only inhibits the interaction of Baz with aPKC itself, whereas Baz also associates with the complex by binding to PAR-6. Although the region of Baz that binds PAR-6 has not been mapped, the first PDZ domain of mammalian PAR-3 binds to the PDZ domain of PAR-6, which is the same region that interacts with Sdt/Pals1 and Crumbs (Hurd et al., 2003; Joberty et al., 2000; Kempkens et al., 2006; Lemmers et al., 2004; Lin et al., 2000; Wang et al., 2004b). This suggests a model in which the Crumbs complex excludes Baz from the PAR-6/aPKC complex by competing for binding to this region of PAR-6.

**Figure 6:**
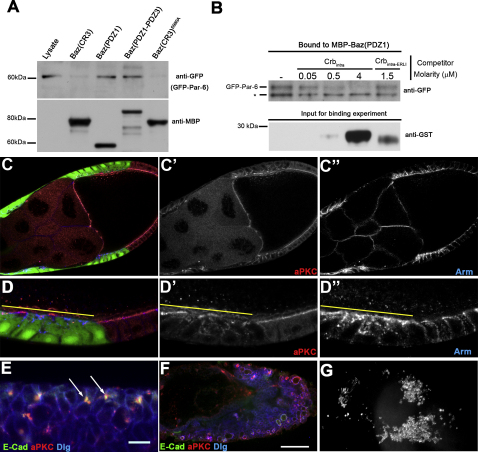
Crumbs Disrupts the Bazooka-Par-6 Interaction (A) The first PDZ domain of Baz is sufficient to pull down PAR-6 from embryonic extracts. MBP-Baz (CR3), MBP-Baz(PDZ1), MBP-Baz (PDZ1-PDZ3), and MBP-Baz (CR3)^S980A^ were incubated with embryonic extracts from PAR-6-GFP-expressing embryos. Input lysate and bound fractions were immunoblotted with α-GFP and α-MBP antibodies. (B) MBP-Baz (PDZ1) was incubated with embryonic extracts from PAR-6-GFP-expressing embryos to which the indicated concentrations of GST-Crb_intra_ or Crb_intra-ΔERLI_ were added. Bound fractions were immunoblotted with α-GFP, whereas input extract/competitor solutions were immunoblotted with α-GST. Low concentrations of GST-Crb_intra_ dramatically reduce PAR-6 binding to Baz(PDZ1), whereas 1.5 μM Crb_intra-ΔERLI_ does not significantly affect PAR-6 binding. (^∗^ marks a nonspecific band whose levels do not vary.) (C and D) Flpout clones overexpressing Baz-PAR-6 marked by GFP expression (green, yellow line in D) and stained for aPKC (red, C′, and D′) and Arm (blue, C″, and D″). (E) Close up of a region of the ectoderm of a stage 9 embryo expressing Baz-PAR-6 zygotically under the control of matα4Gal4-VP16, stained for aPKC (red), E-Cadherin (green), and Dlg (Blue). aPKC and E-Cadherin colocalize in the disorganized epithelium. (F) A late-stage Baz-PAR-6 embryo showing epithelial cyst-like structures. (G) A cuticle preparation of a Baz-PAR-6 embryo.

We analyzed Baz binding to PAR-6 by testing the ability of beads coupled to all three Baz PDZ domains fused to Maltose-binding protein (MBP) or just MBP-Baz PDZ1 to pull down GFP-PAR-6 from embryonic extracts. Both BazPDZ1-3 and BazPDZ1 pulled down GFP-PAR-6 from the extract, whereas the CR3 domain did not, even when the aPKC phosphorylation site was mutated (Figure 6A). Thus, Baz interacts with PAR-6 through its first PDZ domain, as is the case in mammals (Lin et al., 2000). We then tested whether the Crb intracellular domain competes with BazPDZ1 for binding to GFP-PAR-6. Increasing concentrations of Crb_intra_ progressively inhibited the interaction between GFP-PAR-6 and BazPDZ1, whereas Crb_intra_ lacking its last four amino acids (ERLI) did not (Figure 6B). Thus, Crb competes with Baz for binding to PAR-6, and this depends on its C terminus, which functions as a PDZ-binding motif.

If Crb blocks the apical localization of Baz by preventing its binding to PAR-6, a construct in which Baz is constitutively associated with PAR-6 should phenocopy Baz^S980A^. To test this, we used a transgene in which Baz and PAR-6 are directly linked in the same fusion protein (Wirtz-Peitz et al., 2008). Overexpression of Baz-PAR-6 in the follicle cells produces the same phenotypes as BazS980A:GFP and *crb* mutants. The follicle cells often fail to move posteriorly to cover the oocyte, undergo apical constriction, and accumulate Arm apically (Figures 6C and 6D). Expression of Baz-PAR-6 in the embryo with the matα4Gal4:VP16 driver and a paternally derived transgene is lethal. The epithelial structures of these embryos become highly disorganized after gastrulation and show junctional aggregates, in which aPKC colocalizes with AJ markers (Figures 6E). Finally, late-stage Baz-PAR-6 embryos contain epithelial cysts with internal apical lumens, forming disorganized grains of cuticle (Figures 6F and 6G). These results demonstrate that both Crumbs and Baz phosphorylation are required to exclude Baz from the PAR-6/aPKC complex, because the former prevents Baz binding to PAR-6 and the latter blocks its binding to aPKC.

### aPKC Phosphorylation of Baz Is Not Required in Neuroblasts or Oocytes

**Figure S4:**
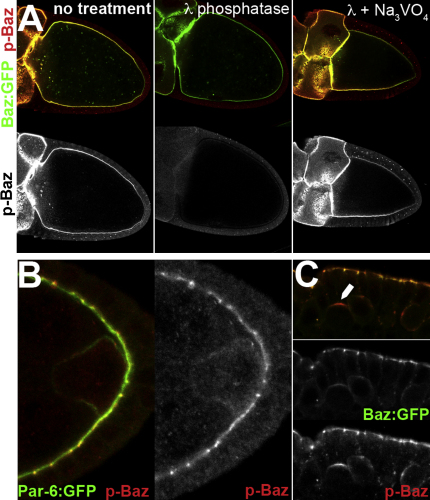
Baz Is Phosphorylated on Ser980 in the Germline and in Neuroblasts, Related to Figure 7 (A) aPKC phosphorylates Baz in the germline. Baz^WT^:GFP overexpressing ovaries were subjected to (-) no treatment, or treated with lambda phosphatase (λ), or lambda phosphatase and phosphatase inhibitor (λ + Na_3_VO_4_), and stained for phospho-Baz (red and lower panels). (B) A stage 6 egg chamber expressing genomic Par-6:GFP (green) (Wirtz-Peitz et al., 2008) was stained for phospho-Baz (red). Endogenous phospho-Baz signal is detected at the cortex of the oocyte, where Par-6:GFP is present. (C) Baz^WT^:GFP phosphorylation at Ser980 is detected in an apical crescent in embryonic neuroblasts (arrow). Baz^WT^:GFP (green) was expressed maternally with matα4GAL4:VP16 and phospho-Baz is shown in red. Separate channels are shown below.

We analyzed whether Baz phosphorylation by aPKC occurs in the female germline and in neuroblasts, where Baz colocalizes with aPKC and PAR-6. Ovaries and neuroblasts expressing Baz^WT^:GFP show strong phospho-Baz staining (Figures S4A and S4C). At endogenous Baz levels, the phosphorylated signal is weaker, but still detectable, at the oocyte cortex, where PAR-6 and aPKC are present (Figure S4B). We therefore examined whether this phosphorylation plays a role in the polarity of either cell type.

**Figure 7:**
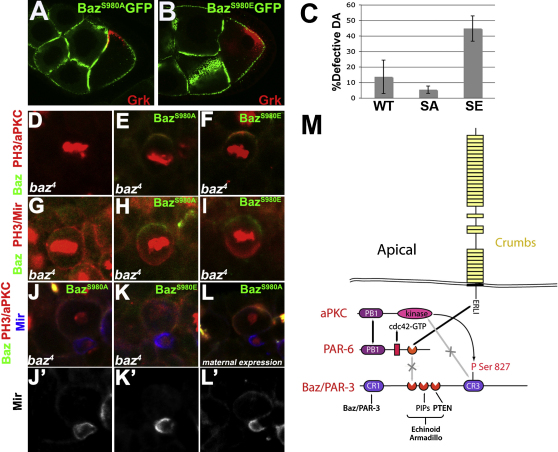
aPKC Phosphorylation Is Not Required in the Oocyte or Neuroblasts (A and B) A stage 9 egg chamber expressing Baz^S980A^:GFP (green) (A) and Baz^S980E^:GFP (green) (B) stained for Gurken (red). (C) Quantification of the Dorsal appendage defects (absence, mislocalization or fusion) of eggs from females expressing Baz^WT^:GFP (WT), Baz^S980E^:GFP (SE), and Baz^S980A^:GFP (SA). The standard deviation is shown for eight independent analyses of about 150 embryos each. (D–I) A metaphase neuroblast in a *baz*^4^ zygotic mutant embryo at stage 11–13 (D and G) and in mutant embryos expressing zygotically Baz^S980A^:GFP (E and H), Baz^S980E^:GFP (F and I) stained for aPKC (red), phospho-Histone 3 (red), and Baz (green) (D–F), and stained for Miranda (red), phospho-Histone 3 (red) and Baz (green) (G–I). (J–L) A telophase *baz*^4^ neuroblast expressing zygotically Baz^S980A^:GFP (J), Baz^S980E^:GFP (K), and maternally Baz^S980A^:GFP (L) stained for aPKC (red), phospho-Histone H (red) and Miranda (blue and J′). (M) A model showing the mechanism of Baz exclusion from the PAR-6/aPKC complex by aPKC phosphorylation and Crb competition for PAR-6 binding. See also Figure S4.

Overexpression of Baz^WT^:GFP or Baz^S980A^:GFP in the female germline has no significant effect on oocyte polarity (Figure 7A and data not shown). However, expression of Baz^S980E^:GFP causes a defect in the localization of the oocyte nucleus and associated Gurken protein, which are often not localized to the dorsal-anterior corner of the cell (31% stage 9 oocytes, n = 52 and 56% stage 10–11, n = 41, Figure 7B). As a consequence, Gurken is not secreted from the dorsal/anterior corner, leading to the production of a high frequency of eggs with defective dorsal appendages (Figure 7C). The fact that expression of Baz^S980A^ has no effect on oocyte polarity suggests that Baz phosphorylation by aPKC is not essential in the female germline, whereas the dominant effect of Baz^S980E^ suggests that the interaction between Baz and aPKC is important in this context.

We addressed the role of Baz phosphorylation in the neuroblasts by examining if the Baz variants could rescue the neuroblast phenotypes of *baz*^4^ homozygous embryos (Atwood et al., 2007). *baz*^4^ zygotic mutants fail to localize aPKC apically (81% defective, n = 37) and as a consequence frequently fail to restrict Miranda (51% defective, n = 51) to the basal cortex (Figures 7D and 7G). Zygotic expression of Baz^S980A^:GFP in *baz^4^* mutant neuroblasts completely rescues the apical localization of aPKC (100%, n = 36) and the basal restriction of Miranda (94%, n = 35), which segregates normally into the GMC during the asymmetric cell division (Figures 7E, 7H, and 7J). Furthermore, Baz^S980E^:GFP also rescues aPKC (96%, n = 26) and Miranda localization (83%, n = 29) in almost all cells (Figures 7F, 7I, and 7K). Maternal overexpression of Baz^S980A^ also allows the segregation of aPKC and Miranda during neuroblast asymmetric cell division, even though epithelial organization is completely disrupted (Figure 7L). Thus, aPKC phosphorylation of Baz appears to be dispensable for neuroblast polarity.

## Discussion

Baz/Par-3, aPKC and PAR-6 are required for the polarization of many different cell types, leading to the assumption that they always form a complex. There is increasing evidence, however, that this complex behaves differently in epithelia. Our results confirm this view, since the majority of Baz in the follicular epithelium localizes independently of aPKC and PAR-6, and below them at the level of the apical junction. Furthermore, the Baz^S980E^ mutant, which cannot interact with aPKC, can rescue the function of Baz in both the follicle cells and embryonic epithelia. Thus, the canonical PAR complex does not appear to be required in the establishment or maintenance of epithelial polarity, at least in *Drosophila*.

Baz fails to associate with aPKC and PAR-6 in epithelial cells because it is excluded from the complex by the combined action of the Crumbs complex and of Baz phosphorylation on Serine 980 by aPKC. aPKC phosphorylates Baz in the middle of its conserved aPKC-binding domain, CR3, and several lines of evidence indicate that this prevents the binding of Baz to aPKC (Figure 7M). First, Baz only interacts with aPKC in a yeast two-hybrid assay when S980 is mutated to alanine to prevent aPKC phosphorylation (Benton and St Johnston, 2003b). Similarly, the binding of mammalian PAR-3 to PKCζ in a pulldown assay is blocked when the equivalent serine is mutated to the phosphomimetic glutamate (Nagai-Tamai et al., 2002). Second, phosphomimetic and wild-type Baz do not colocalize with aPKC at the apical cortex of the follicle cells, whereas the nonphosphorylatable form does, and this apical localization of BazS980A is aPKC dependent, because it is abolished in *aPKC* mutant clones. Thus, the direct binding of aPKC to Baz can only ever be short lived when aPKC is active, because phosphorylation of S980 will disrupt the interaction.

Baz phosphorylation is not sufficient to prevent its association with PAR-6/aPKC complex, because Baz binds directly to the PDZ domain of PAR-6. However, both Crb and Sdt bind to the same domain of PAR-6, and out-compete Baz for binding when Baz cannot also interact with aPKC (Figure 7M). Since aPKC seems to phosphorylate Baz in all cell types that we have examined, the epithelial-specific exclusion of Baz from the PAR-6/aPKC complex must therefore be determined by the presence of Crb and Sdt, both of which are required specifically in epithelial cells (Wodarz et al., 1995). It is striking that embryonic expression of either Baz^S980A^ or Baz-PAR-6 produces a phenotype that closely resembles that of *crb* and *sdt* null mutants, indicating that the apical exclusion of Baz is a key function of the Crumbs complex.

In nonepithelial cells such as the oocyte or neuroblasts, Baz, aPKC, and PAR-6 define a single cortical domain. The consequence of the apical exclusion of Baz by aPKC phosphorylation and the Crumbs complex is to split this into two adjacent cortical domains, with PAR-6/aPKC marking the apical cortex and Baz, the most apical region of the lateral cortex. This plays a key role in the organization of the epithelium because the Baz domain defines the position of the AJ (Harris and Peifer, 2005). Indeed, Baz has been shown to bind directly to the AJ components Echinoid and Arm (Wei et al., 2005). The Baz variants that are not excluded from the apical domain (Baz^S980A^ and Baz-PAR-6) cause the apical recruitment of Arm and E-Cadherin and lead to the formation of wedge-shaped cells with reduced apical domains and expanded lateral domains. Thus, the apical exclusion of Baz by aPKC and the Crumbs complex restricts the extent of the apical/lateral AJ and defines the border between the apical and lateral domains.

Our results suggest that the apical exclusion of Baz may play an important role in epithelial morphogenesis. First, Baz^S980A^ expression inhibits the cuboidal to columnar transition of the follicle cells and prevents their posterior movement to cover the oocyte. Second, Baz^S980A^ expression only disrupts the epithelial organization of the embryonic ectoderm once the morphogenetic movements of germband extension are underway. This is probably because Baz is localized to the apical/lateral region by a different mechanism during cellularization, which determines where the AJs initially form (Harris and Peifer, 2005). As long as the cells are static, the AJs will tend to stay in place because they are held by homophilic adhesion between adjacent cells, and they therefore anchor Baz in this position. During germband extension, however, the AJs undergo extensive remodeling (Bertet et al., 2004; Blankenship et al., 2006). The mechanisms that positioned Baz during cellularization no longer function at this stage, and the apical exclusion of Baz by aPKC phosphorylation and Crumbs becomes essential to position the AJs and maintain epithelial organization. It is possible that Baz phosphorylation also plays a more active role in driving the cell shape changes, as Baz becomes enriched along the dorsal and ventral cell boundaries as germband extension occurs (Blankenship et al., 2006; Zallen and Wieschaus, 2004).

It has recently been reported that Crb is specifically required in epithelia that are undergoing morphogenetic movements (Campbell et al., 2009; Harris and Tepass, 2008). We propose that this reflects its role in the apical exclusion of Baz to maintain the apical domain during junctional remodeling. Unlike *crb* mutants, BazS980A also disrupts the organization of epithelia that are not changing shape, such as those in the head region of the early embryo. This difference is probably due to the overexpression of Baz. Under normal conditions, almost all Baz remains associated with the AJs, which provide a saturatable scaffold that anchors it to the junctional domain. When BazS980A is overexpressed, the extra protein cannot be anchored at the junctions and goes apically, leading to the apical mislocalization of the AJs and the gradual shrinking of the apical domain.

PAR-3 localizes beneath aPKC and PAR-6 in mammalian epithelia, raising the possibility that it is excluded from the apical domain by the same mechanism as in *Drosophila* and that this also defines the position of the apical junction (Afonso and Henrique, 2006; Martin-Belmonte et al., 2007; Totong et al., 2007). However, the apical junction in vertebrates is the tight junction rather than the AJ. Nevertheless, the available data suggest that aPKC phosphorylation of PAR-3 may perform an analogous role in the positioning of the apical (tight) junction in mammals. First, PAR-3 localizes to the tight junctions as they form and interacts directly with the tight junction components, JAM1-3 and Nectin (Ebnet et al., 2001; Itoh et al., 2001; Kohjima et al., 2002). Second, PAR-3 directs tight junction formation, as overexpression of PAR-3 increases the rate at which tight junctions form, whereas dominant negative PAR-3 and PAR-3 RNAi inhibit tight junction formation (Chen and Macara, 2005). Third, PAR-3 is phosphorylated by aPKC on the same conserved serine in CR3 as Baz to disrupt the PAR-3/aPKC interaction, and nonphosphorylatable PAR-3 disrupts tight junction formation (Nagai-Tamai et al., 2002). Furthermore, a PAR-3 mutant that cannot bind aPKC rescues tight junction formation, just as BazS980E does in *Drosophila* (Horikoshi et al., 2009). Thus, it is possible that the apical/lateral boundary is positioned in the same way in mammals and *Drosophila,* despite the different arrangement of junctions.

One of the most surprising features of our results is that they reveal that Baz performs completely different functions in nonepithelial and epithelial cells. In the neuroblast, for example, Baz acts an aPKC targeting factor by recruiting the PAR-6/aPKC complex to the cortex through the binding of PAR-6 and serves as an aPKC specificity determinant by recruiting Numb to the Baz/PAR-6/aPKC complex, so that aPKC can phosphorylate it (Wirtz-Peitz et al., 2008). By contrast, Baz functions separately from PAR-6 and aPKC in epithelial cells, where its principle function is to stabilize and position the apical junction. This presumably depends on other activities of Baz, such as its binding to Arm and Echinoid and its recruitment of PTEN to regulate Phosphatidylinositide 4,5 P_2_ (PIP2) levels (Pinal et al., 2006; von Stein et al., 2005; Wei et al., 2005; Wu et al., 2007). Thus, Baz appears to have evolved two different sets of functions to polarize epithelial versus nonepithelial cells.

## Experimental Procedures

### *Drosophila* Strains and Genetics

The mutant strains and transgenic lines used in this study are described in the Extended Experimental Procedures. Clonal analyses were performed with the FLP/FRT system using nuclear GFP as marker of wild-type cells (Xu and Rubin, 1993). *baz* transgenic rescue experiments in the follicular epithelium were performed by induction of transgene expression with the AyGal4 system (Ito et al., 1997). Clones were analyzed in the follicle epithelium of *baz^4^, FRT9.2/Ubi-GFP, FRT9.2; AyGal4, UAS:Baz ^transgene^/ hsFlp* flies. The tubulin promoter was used for strong overexpression in FLPout clones. *matα4-GAL4:VP16* V32A was used to induce strong expression of UAS transgenes in the maternal germline. For strong expression in early embryos, embryos laid by *matα4-GAL4:VP16/Baz^transgene^* mothers were analyzed. Rescue experiments in *baz^4^* zygotic mutants were performed by analysis of Y/*baz^4^*; *matα4-GAL4:VP16/UAS:Baz^trangene^* embryos with a maternally derived GAL4 driver and paternally derived transgenes. This approach produces weaker expression during early embryogenesis.

### Immunological Methods

A phosphospecific antibody against phosphoserine 980 of Baz was raised in rabbits by injection of the phosphorylated peptide CHFSRDALGRR{pSer}ISE and subsequent immunodepletion with the unmodified peptide and affinity purification with the phosphorylated peptide (Genscript, New Jersey). Immunofluorescence and western blotting were performed using the primary antibodies at dilutions listed in the supplemental data with the appropriate combination of mouse, rabbit and rat FITC, Cy5 or Texas Red secondary antibodies (Jackson ImmunoResearch Laboratories). Actin was visualized with Rhodamine-conjugated phalloidin (Invitrogen).

### Molecular Biology

pBluescript subclones containing the wild-type Bazooka cDNA (Kuchinke et al., 1998) were used as templates to generate Baz^S980A^ and Baz^S980E^ by oligonucleotide directed mutagenesis according to the manufacturer's protocol (Stratagene). Details are described in the Extended Experimental Procedures. For expression of MBP-fusion proteins, amino acids 251–450 (PDZ1), 251–750 (PDZ1-PDZ3), and 829–1168 (CR3) from wild-type Baz or Baz S980A were cloned into a pMAL vector (New England Biolabs). The GST-Crb_intra_ and GST-Crb_intra-ΔERLI_ expression constructs were obtained from E. Knust (Kempkens et al., 2006). Recombinant GST and MBP proteins were purified accordingly to the manufacturer's protocols.

### Biochemical Analysis

Ovaries expressing the different GFP-tagged Baz transgenes driven by Cy2Gal4 were homogenized in Lysis buffer (125 mM NaCl, 50 mM Tris-HCl [pH 7.5], 5% Glycerol, 1 mM MgCl_2_,1 mM EDTA, 0.2% NP-40, 0.5 mM DTT, phosphatase inhibitor cocktail 1 [Sigma], and Protease Inhibitor cocktail [Roche]). GFP-tagged proteins were immunoprecipitated with an affinity-purified sheep polyclonal anti-GFP antibody bound to magnetic Dynabeads (Invitrogen), washed three times with lysis buffer, and eluted with HCl/Glycine (pH 2.5). For Kinase assays, embryonic extracts from *par-6^D226^* par-6 > Par-6:GFP embryos (Wirtz-Peitz et al., 2008) were used for immunoprecipitation as described above. Kinase reactions were assembled directly on the immunoprecipitated Par-6:GFP beads in kinase buffer (250 mM HEPES [pH 7.4], 0.2 mM EDTA, 1% glycerol, 150 mM NaCl, 10 mM MgCl_2_) and 3 μg purified MBP:Baz fusion proteins were added as substrates. Reactions were initiated by addition of ATP mix (1.5 μl vol 1 mM ATP, 1 μl γ-^32^P-ATP [5 mCi/ml]), and incubated at 30°C for 25 min. After the incubation, beads were removed and samples were boiled in SDS-PAGE sample buffer. For protein-binding assays, 25 μg of the purified MBP-fusion proteins bound to amylose resin (NEB) were incubated with 2 mg protein extracts from *par-6^D226^*, par-6 > Par-6-GFP embryos (Wirtz-Peitz et al., 2008). After extensive washing with binding buffer (50 mM Tris-HCl [pH 7.5], 150 mM NaCl, 1 mM EDTA, 0.2% NP-40), bound proteins were eluted with 10 mM Maltose in binding buffer. So that competition could be tested, variable concentrations of GST-fusion Crumbs proteins were added to the embryonic extract prior to pull-downs with MBP-BazPDZ1. Anti-GST (Sigma) and anti-MBP (NEB) were used to detect fusion proteins by immunoblotting.

**Extended Experimental Procedures**

***Drosophila* Strains**

*w^1118^* was used as control. The following mutant alleles were used and are null or strong mutations: *crb^2^* (Tepass et al., 1990); *sdt^XP96^* (Muller and Wieschaus, 1996); *baz^4^* (Muller and Wieschaus, 1996); *aPKC^K06403^* (Wodarz et al., 2000); *par-6^Δ226^* (Petronczki and Knoblich, 2001). The following UAS transgenes were used: *UASp:BazWT-GFP* (Benton and St Johnston, 2003b), *UASt:Crb^WT^* (Wodarz et al., 1995) and *UASt:Baz-Par-6* (Wirtz-Peitz et al., 2008), *UASp:Baz^S980A^-GFP* and *UASp:Baz^S980E^-GFP* (this study). Chromosomes containing *UASt:Crb^WT^* recombined with *UASp:Baz* transgenes were generated for co-expression experiments. *Cy2-GAL4* (Queenan et al., 1997) was used to drive expression in the follicular epithelium from stage 8 of oogenesis onward. The AyGal4 system used for rescue experiments in the follicle epithelium contains an Act5c promoter that is interrupted by a FRT cassette, which must be excised to induce expression of transgenes (Ito et al., 1997). To carry out the rescue experiments, FLP recombinase was expressed under the control of the heat-shock promoter to generate the mutant clones, and also to excise the FRT cassette from the GAL4 construct. In order to generate FlpOut clones expressing at very high levels UAS transgenes were crossed into y,w, *hsFlp; tub-FRT-cc-FRT-Gal4, UAS:GFP*, and progeny were heat shocked during third larval instar and pupal stages.

**Generation of Bazooka Transgenic Lines**

Baz^S980A^ was generated using 5′ CTTTGGGACGACGCGCCATCTCTGAGAAGC 3′ and 5′ GCTTCTCAGAGATGGCGCGTCGTCCCAAAG 3′ primers and Baz^S980E^ with 5′ CTTTGGGACGACGCGAGATCTCTGAGAAGC 3′ and 5′ GCTTCTCAGAGATCTCGCGTCGTCCCAAAG 3′. The mutated Baz coding region was amplified by PCR, sequenced and subcloned in between the KpnI and SpeI sites of the pUASp mGFP6 vector (Rorth, 1998). pUASp:Baz^S980A^:GFP and pUASp:Baz^S980E^:GFP transgenes were introduced into flies by standard germline transformation techniques.

**Specificity Test of Phospho-Baz^S980^ Antibody**

For immunofluorescence (IF), samples were treated after fixation with 400U of lambda phosphatase (New England Biolabs) or lambda phosphatase plus 10 mM of its inhibitor, Na_4_VO_3,_ for 1hr at 30°C in the buffer supplied. For Western blotting (WB) experiments, ovary protein extracts were treated as above and then boiled in SDS-PAGE sample buffer.

**Primary Antibodies and Dilutions Used**

Rabbit p-Baz (1/100 IF and 1/1000 WB), rabbit anti-PKCz (1/500 IF and 1/2000 WB, (C-20 Santa Cruz)), rabbit anti-Miranda (1:1000, (Ikeshima-Kataoka et al., 1997)) and guinea-pig anti-Miranda (1/500, kindly provided by Kate Beckingham), rat anti-DE-Cad (1/20 (Oda et al., 1994)) mouse anti-Gurken (1/30, Developmental Studies Data Bank (DSHB), University of Iowa), mouse anti-Dlg (1/200, DSHB), rabbit anti-Lgl (1/500 (Betschinger et al., 2003)), rabbit anti-Par-6 (1/500 (Petronczki and Knoblich, 2001)), mouse anti-Arm (N2-7A1, 1/100, DSHB), rabbit anti-Baz (1/1000 IF and 1/5000 for WB) (Wodarz et al., 1999), mouse anti-Crumbs (Cq4, 1/50, DSHB), rabbit anti-D-Patj 1/500 (Tanentzapf et al., 2000), rabbit anti-p-H3 (1/500; Cell Signaling) and mouse anti-α-tubulin (1/2000, DM1A, Sigma).

**Imaging**

Imaging of fixed samples was performed using a Zeiss LSM510 scanning laser confocal microscope (Carl Zeiss MicroImaging, Inc.) with 40x oil lenses (Plan-NeoFluor; NA 1.3) and LSM510 AIM software. Images were processed using ImageJ and Adobe Photoshop.

For time-lapse imaging, embryos at the end of cellularization were mounted in Voltalef oil (Attachem) and frames were taken every 30-60 s on an Olympus FV1000 inverted confocal microscope with 40 x oil lenses (Oil UPlan FLN, NA1.3). Movies were then processed with ImageJ.

## Supplemental Information

Movie S1. Related to Figure 4 A 3D projection of the epidermal surface of embryos expressing Baz^WT^:GFP (left) and Baz^S980A^:GFP (right) driven by matα4Gal4VP16.

Movie S2. Related to Figure 4 Time-lapse movies of embryos expressing Baz^WT^:GFP (top) and Baz^S980A^:GFP (bottom).This file encompasses three movies showing sequentially lateral views, the ventral surface, or the anterior region of embryos expressing the different transgenes. The first movie shows lateral views of developing embryos expressing Baz^WT^:GFP and Baz^S980A^:GFP. Transmitted light images are shown on the left to indicate the developmental stage of the embryos, and the right channel shows the Baz:GFP fluorescence. The movie begins during the final stages of cellularization and extends until the slow phase of germ band elongation (timings are given relative to the start of gastrulation, which is set as 0 min). The second movie shows a ventral view, showing that the ventral furrow (t = 0) forms normally in Baz^S980A^:GFP overexpressing embryos, but the epithelium starts to become disorganized soon afterward, during the fast phase of germband elongation. Cells undergoing division are labeled with an RFP-tagged microtubule-binding protein. The third movie shows an anterior region of a Baz^S980A^:GFP-expressing embryo. The cephalic furrow forms at t = 15–20 min and marks the boundary between the head and the trunk. Junctional aggregates of Baz^S980A^:GFP form in the trunk and in the head region anterior to the cephalic furrow. The scale bar represents 20 μm

Document S1. Article Plus Supplemental Information
