# Supplementary figures and images for: aPKC Phosphorylation of Bazooka Defines the Apical/Lateral Border in Drosophila Epithelial Cells
Source: Cell. 2010 Apr 30;141(3):509–23. doi: 10.1016/j.cell.2010.02.040 (PMC2885938; doi:10.1016/j.cell.2010.02.040)

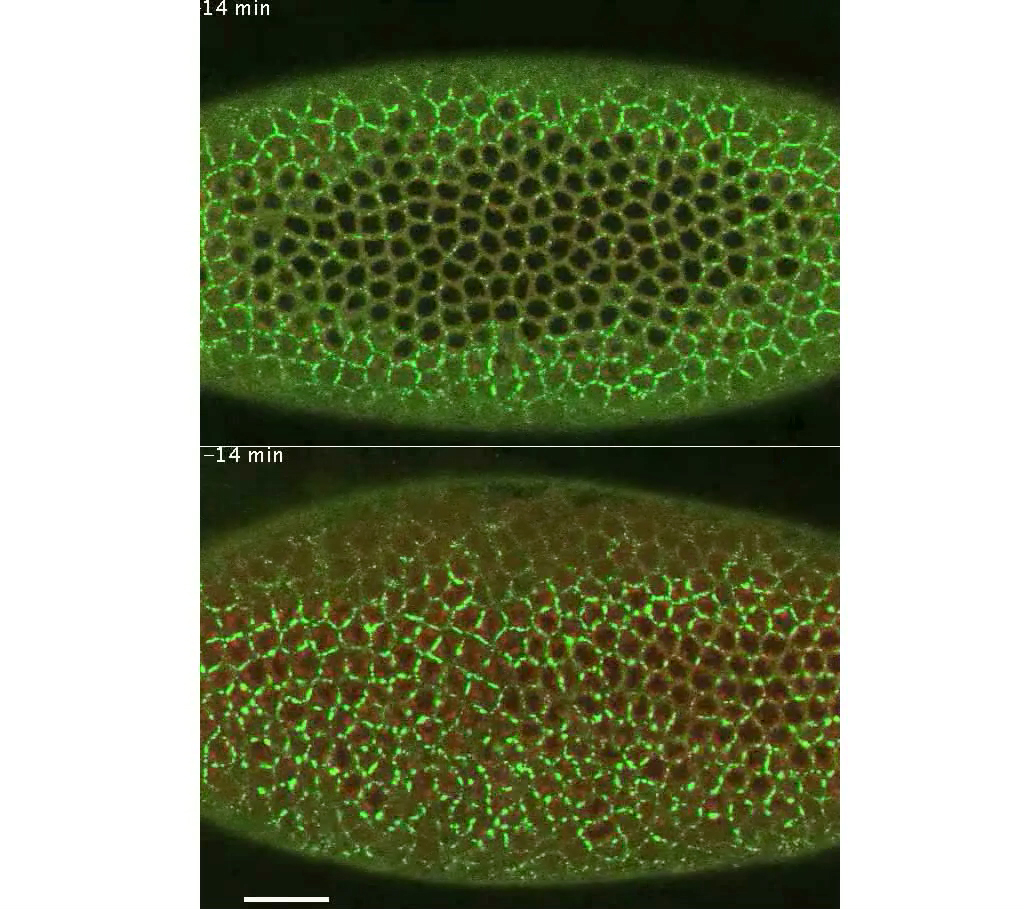

Supplement: Movie S2. Related to Figure 4 — Time-lapse movies of embryos expressing BazWT:GFP (top) and BazS980A:GFP (bottom).This file encompasses three movies showing sequentially lateral views, the ventral surface, or the anterior region of embryos expressing the different transgenes. The first movie shows lateral views of developing embryos expressing BazWT:GFP and BazS980A:GFP. Transmitted light images are shown on the left to indicate the developmental stage of the embryos, and the right channel shows the Baz:GFP fluorescence. The movie begins during the final stages of cellularization and extends until the slow phase of germ band elongation (timings are given relative to the start of gastrulation, which is set as 0 min). The second movie shows a ventral view, showing that the ventral furrow (t = 0) forms normally in BazS980A:GFP overexpressing embryos, but the epithelium starts to become disorganized soon afterward, during the fast phase of germband elongation. Cells undergoing division are labeled with an RFP-tagged microtubule-binding protein. The third movie shows an anterior region of a BazS980A:GFP-expressing embryo. The cephalic furrow forms at t = 15–20 min and marks the boundary between the head and the trunk. Junctional aggregates of BazS980A:GFP form in the trunk and in the head region anterior to the cephalic furrow. The scale bar represents 20 μm [file mmc2.jpg]
